# Transanal endoluminal repair for anastomotic leakage after low anterior resection

**DOI:** 10.1186/s12893-022-01484-4

**Published:** 2022-01-26

**Authors:** Yi-Chang Chen, Yuan-Yao Tsai, Tao-Wei Ke, Abe Fingerhut, William Tzu-Liang Chen

**Affiliations:** 1 Department of Colorectal Surgery, China Medical University Hospital, Taichung, Taiwan; 2 Department of General Surgery, Ruijin Hospital, Shanghai Jiao Tong University School of Medicine, Shanghai Minimally Invasive Surgery Center, Shanghai, 200025 People’s Republic of China; 3 Medical University Hospital of Graz, Graz, Austria; 4 China Medical University Hsinchu Hospital, Zhubei, Taiwan

**Keywords:** Transanal endoluminal repair, Colorectal anastomosis, Anastomosis leakage, Low anterior resection

## Abstract

**Background:**

There is still no consensus on the management of colorectal anastomotic leakage after low anterior resection. The goal was to evaluate the outcomes of patients who underwent transanal endoluminal repair + laparoscopic drainage ± stoma vs. drainage only ± stoma.

**Methods:**

Retrospective chart review of patients sustaining anastomotic leakage after laparoscopic low anterior resection between January 2013 and September 2020 who required laparoscopic reoperation.

**Results:**

Forty-nine patients were included, 22 patients underwent combined laparoscopy and transanal endoluminal repair and 27 patients had drainage with a stoma (n = 16) or drainage alone (n = 11), without direct anastomotic repair. The overall morbidity rate was 30.6% and the mortality rate was 2%. Combined laparoscopic lavage/drainage and transanal endoluminal repair of anastomotic leakage was associated with a lower complication rate (13.6% vs. 44.4%, p = 0.03) and fewer intraabdominal infections (4.5% vs. 29.6%, p = 0.03) compared with no repair.

**Conclusions:**

Combined laparoscopic lavage/drainage and transanal endoluminal repair is effective in the management of colorectal anastomosis leakage and was associated with lower morbidity—in particular intraabdominal infection—compared with no repair. However, our results need to be confirmed in larger, and ideally randomized, studies.

## Introduction

Anastomotic leakage is one of the most dreaded complications after elective colorectal surgery and is associated with high morbidity, mortality and poor oncological outcome [1–4]. Despite well-recognized preventive measures, the overall leakage rate ranges from 1 to 22%, while extra-peritoneal anastomosis leakage rates range from 3 to 19% [5–7]. Treatment strategies differ according to whether the leak is extra-peritoneal or intra-peritoneal [8]. Traditionally, extra-peritoneum anastomosis failure calls for resection with end stoma creation, but which is never reversed in 12–56% of patients [8]. Recently there has been a paradigm shift from resection to preservation of the anastomosis [8–10]. The advantage of anastomotic preservation is to avoid an additional resection, the risk of anastomotic leakage when intestinal continuity is finally re-established, and the fact that many patients with the Hartmann procedure do not have stoma reversal [11].

We have previously reported that combined repeat laparoscopy and transanal endoluminal anastomosis repair after colorectal anastomosis leakage was safe and feasible [12, 13]. However, to the best of our knowledge, no studies have compared outcomes of patients who undergo laparoscopic anastomotic repair in comparison to those who are treated by simple drainage and stoma, the so-called “divert and drain” technique [8]. The goal of our study is to compare re-laparoscopic lavage/draingage + transanal endoluminal repair ± stoma vs. drainage ± stoma.

## Materials and methods

This observational study was a retrospective chart review of all consecutive patients with anastomotic leakage after laparoscopic low anterior resection who underwent laparoscopic re-intervention between January 2013 and September 2020 at single tertiary center, China Medical University Hospital, Taichung, Taiwan.

Peritoneal contamination and collections were evaluated by abdominal computed tomographic (CT) scan and laparoscopy. Anastomotic leaks were defined clinically or identified during endoscopic/radiologic examination, abdominal CT [12] and confirmed by digital examination before re-intervention at the same time as evaluation of the defect size. Acute Physiology And Chronic Health Evaluation II (APACHE II) score was used to evaluate sepsis severity before the second operation.

Laparoscopic re-intervention was indicated when patients had symptomatic leaks including fever, peritonitis, unstable clinical status, and/or failure of conservative treatment. The Hartmann procedure was indicated when bowel tissue was not viable, anastomosis dehiscence > 50% of the circumference, associated with unstable clinical status. The decision to preserve the anastomosis and/or transanal endoluminal repair or create a stoma was taken according to surgeon experience and preference.

## Surgical technique

The patients were placed in Trendelenburg position. The open technique was used to insert a 10 mm port through the previous umbilical port site to create the pneumoperitoneum and then insert the camera. Pneumoperitoneum pressure was maintained less than 12 mm Hg. All other 5 mm working ports were inserted via previous trocar wounds. Diagnostic laparoscopy was performed to visualize the entire abdominal cavity. After initial evaluation, blunt adhesiolysis with the suction irrigator and occasional sharp dissection using monopolar scissors or another energy-driven device was performed. All four quadrant were copiously lavaged with saline to eliminate all intra-abdominal collections.

**Fig. 1:**
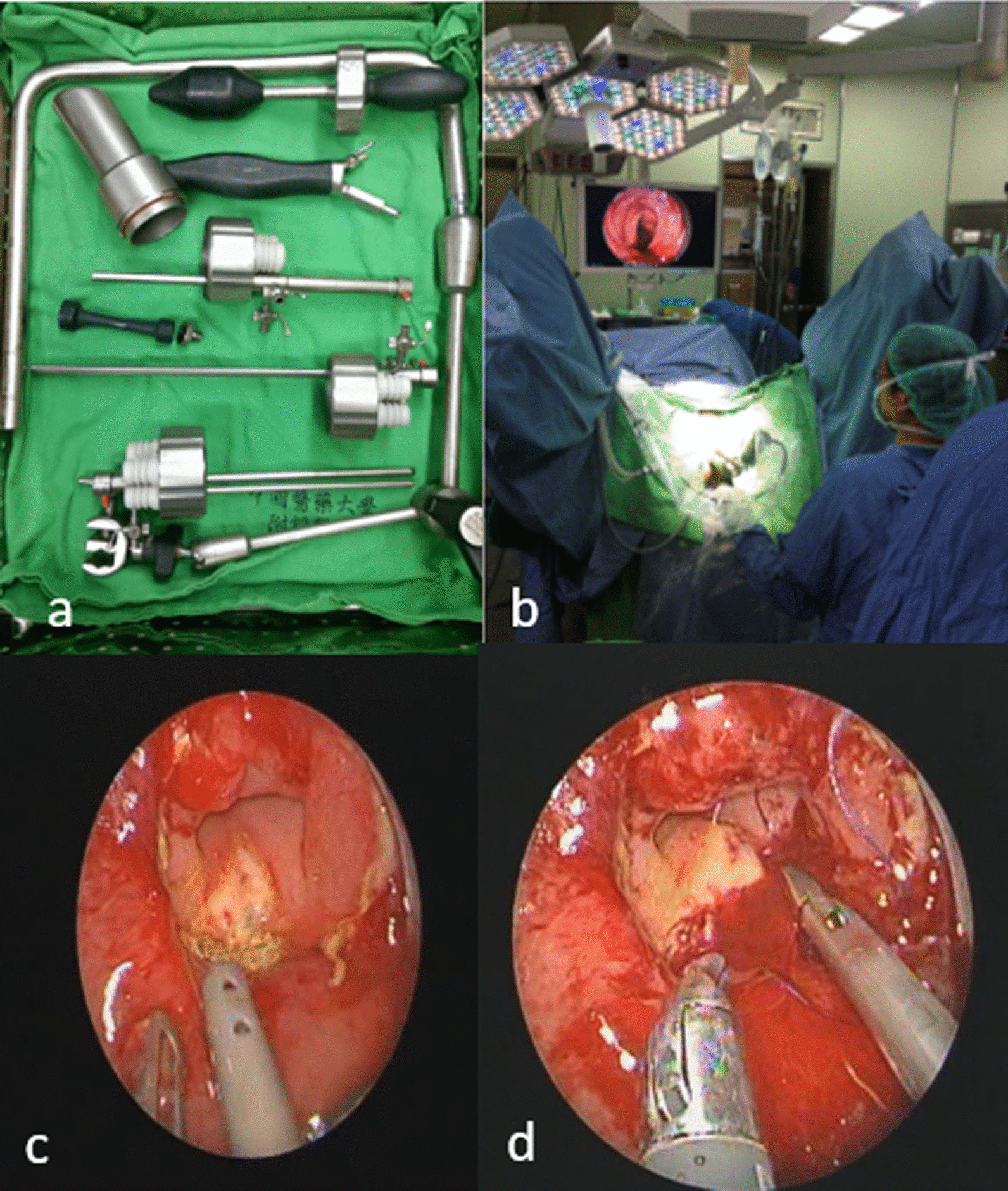
Transanal endoluminal repair.** a** TEO platform.** b** Patient was under lithotomy position.** c** Irrigation over leakage site.** d** Transanal endoluminal repair with 3.0 Vicryl

After laparoscopic exploration, the defect size was confirmed by colonoscopy or transanal endoluminal examination. Transanal endoluminal repair of the dehiscence site was attempted with a TEO platform, as described previously [13] (Fig. 1). By direct transanal endoluminal examination, we were able to confirm the leak, assess the size of the dehiscence, the distance from the anal verge, and the vascular aspect of the mucosa of the adjacent colorectal segments (Fig. 1). The anastomotic leak site was closed with interrupted Vicryl® 2-0 sutures (Fig. 1). The repair was visualized via the TEO or flexible colonoscopy, and tested for air-tightness. A diverting colostomy was performed in all patients who did not already have one during the initial operation (except one patient in the repair group).

Anastomotic healing was evaluated by digital examination every week during the 1st month and then flexible sigmoidoscopy every 3 months. Lastly, lower gastrointestinal imaging was performed before stoma closure.

Morbidity and mortality were recorded at 30 days after the second operation or during the same hospital stay. Surgical site infection (SSI) or organ space SSI was diagnosed by CT scan. Prolonged ileus was defined as absence of passage of flatus/stools > 7 days.

Permanent stoma was defined as persistence of stoma > 1 year after operation or at the time of death [14].

Categorical data are presented as numbers (percentages) and were compared with the Chi-square or Fisher’s exact test, as appropriate. Continuous data are expressed as means for normally distributed variables, compared with the Student *t*-test or medians with ranges for non-parametric data and were compared with the Mann–Whitney U test.

All tests were two-sided and p values less than 0.05 were considered to be statistically significant. All statistical analyses were performed with SPSS for Windows (version 19.0; IBM-SPSS Inc., Armonk, NY).

## Results

### Patient characteristics

**Fig. 2:**
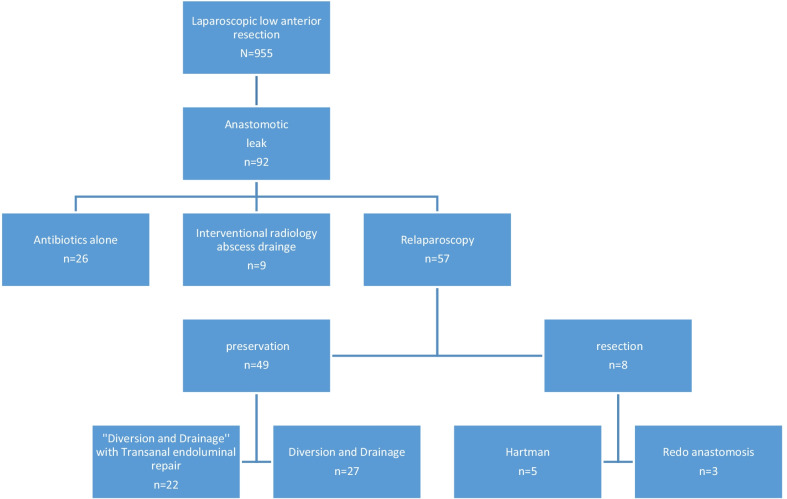
Patient population flow chart

Between January 2013 and September 2020, 955 consecutive patients underwent laparoscopic low anterior resection with anastomosis in our Institution: 92 patients (9.6%) were recorded as having an anastomotic leakage: 26 patients were treated conservatively, nine underwent percutaneous abscess drainage while 57 had laparoscopic reintervention (Fig. 2). Of the latter, the anastomosis was preserved in 49 patients while eight patients required resection (3 redo anastomoses and 5 Hartmann operations). The 49 patients with preservation constitute our study population: 22 underwent transanal endoluminal repair while 27 patients (control group) had drainage alone (n = 11) or with a stoma (n = 16), but no direct anastomotic repair. Patient baseline characteristics and details of the initial operation are indicated in Table 1.

**Table 1:** Baseline characteristics and details of first operation

	All (n = 49)	Endoluminal repair (n = 22)	No repair (n = 27)	P value
Age	60 (32–89)	56 (32–87)	66 (32–89)	0.10
BMI	23.1 (17.3–31.1)	24.1 (17.7–30.1)	22.0 (17.3–30.0)	0.08
Gender				0.26
Male	39 (79.5%)	17 (77.3%)	22 (81.5%)	
Female	10 (20.5%)	5 (22.7%)	5 (18.5%)	
ASA				0.31
2	33 (67.3%)	18 (81.8%)	15 (55.6%)	
3	16 (32.7%)	4 (18.2%)	12 (44.4%)	
Malignant	47 (95.9%)	20 (90.9%)	27 (100%)	0.24
Tumor site				0.17
Sigmoid	5 (6.3%)	2 (9.1%)	3 (11.1%)	
Rectosigmoid	9 (18.3%)	2 (9.1%)	7 (25.9%)	
Upper rectum	11 (22.4%)	4 (18.2%)	7 (25.9%)	
Middle rectum	12 (26.5%)	6 (27.3%)	6 (22.2%)	
Lower rectum	12(26.5%)	8 (36.4%)	4 (14.8%)	
Stage				0.68
I	7 (14.2%)	4 (18.18%)	3 (11.11%)	
II	10 (20.4%)	4 (18.18%)	6 (22.22%)	
III	20 (40.8%)	9 (40.91%)	11 (40.74%)	
IV	10 (20.4%)	3 (13.64%)	7 (25.93%)	
Neoadjuvant therapy	11 (22.4%)	4 (18.18%)	7 (25.9%)	0.55
Stoma formation				0.23
1st operation	23(46.9%)	12 (54.5%)	11 (40.7%)	
Anastomotic method				0.23
Stapled	45 (91.8%)	20 (90.9%)	25 (92.5%)	
Handsewn	4 (8.2%)	2 (9.1%)	2 (7.5%)	
Distance of anastomosis from anal verge	5 (0–18)	4 (0–12)	6 (0–18)	0.61

*BMI* body mass index; *ASA* American Society of Anesthesiologists

### Immediate peri-laparoscopic reintervention outcome (Table 2)

**Table 2:** Patient characteristics before re-laparoscopy

	All (n = 49)	Endoluminal repair (n = 22)	No repair (n = 27)	
APACHE II score	8 (3–23)	7 (3–20)	8 (4–23)	0.13
Interval to detection of anastomotic leak (days)	5 (1–17)	4 (1–13)	4 (1–17)	0.54
Anastomotic defect				0.76
Total	4 (8.3%)	2 (9.09%)	2 (7.4%)	
½ circumference	6 (12.2%)	4 (18.18%)	2 (7.4%)	
$${\raise0.7ex\hbox{$1$} \!\mathord{\left/ {\vphantom {1 3}}\right.\kern-\nulldelimiterspace} \!\lower0.7ex\hbox{$3$}}$$ circumference	19 (38.7%)	8 (36.36%)	11 (40.7%)	
¼ circumference	20 (40.8%)	8 (36.36%)	12 (44.4%)	
Peritoneal contamination				0.47
Local	33 (67.3%)	16 (72.7%)	17 (62.9%)	
Diffuse	16 (32.7%)	6 (27.3%)	10 (37.1%)	

Median APACHE II score of all patients was 8 with no statistically significant difference between the two groups. Three patients (13.6%) in the repair group required conversion to open surgery (one due to inadequate exposure and two because of dense adhesions). One patient (3.7%) in the control group was converted to laparotomy due to diffuse oozing of pelvic rough surface that was successfully controlled by compression after conversion. One patient in the repair group did not have stoma formation. The 30 day complication rate was statistically significantly lower in the repair group [3/22 patients (13.6%) vs. 12/27 patients (44.4%)] in the control group, p = 0.03). The intraabdominal infection rate was lower in the repair group (4.5% vs. 29.6%, p = 0.03). The median hospital stay after the second operation was 10 (1–62) days; the difference between the two groups was not statistically significant.

### Complications and additional interventions related to the second operation (Tables 3 and 4)

**Table 3:** Intraoperative features and postoperative course after re-laparoscopy with anastomotic preservation

	All (n = 49)	Endoluminal repair (n = 22)	No repair (n = 27)	
Blood loss units (cc)	0 (0–6650)	0 (0–30)	0 (0–6650)	0.27
Stoma created during re-operation	25 (51.0%)	9 (40.9%)	16(59.3%)	
No stoma	1 (2.1%)	1 (4.6%)	0	
Conversion	4 (8.1%)	3 (13.6%)	1 (3.7%)	1.0
Post-operative complications	15 (30.6%)	3 (13.6%)	12 (44.4%)	0.03
Ileus	4 (8.1%)	0	4 (14.8%)	0.11
SSI	4 (8.1%)	2 (9.0%)	2 (7.4%)	1.0
Deep OS/SSI	9 (18.3%)	1 (4.5%)	8 (29.6%)	0.03
Anastomotic stricture	2 (4.0%)	1 (4.5%)	1 (3.7%)	1.0
Persistent purulent discharge	3 (6.1%)	2 (9.0%)	1 (3.7%)	0.58
Gastric ulcer	1 (2.0%)	0	1 (3.7%)	1.0
Atelectasis	1 (2.0%)	0	1 (3.7%)	1.0
Death	1 (2.0%)	0	1 (3.7%)	1.0
Additional intervention (after second operation)	8 (16.3%)	3 (13.6%)	5 (18.5%)	1.0
Surgical	6 (12.2%)	3 (13.6%)	3 (11.1%)	
Non surgical	2 (4.0%)	0	2 (7.4%)	
Median duration of hospital stay (days)	9.5 (1–62)	11 (1–45)	9 (3–62)	0.68
Permanant stoma	10 (20.4%)	4 (18.2%)	7 (25.9%)	0.73
Incisional hernia	3 (6.1%)	2 (5.9%)	1 (6.7%)	0.71

*SSI* surgical site infection; *OS/SSI* organ space surgical site infection

**Table 4:** Post-operative course after second operation

	All (n = 49)	Endoluminal repair (n = 22)	No repair (n = 27)
Additional intervention	8 (16.3%)	3 (13.6%)	5 (18.5%)
Surgical complications	6 (12.2%)	3 (13.6%)	3 (11.1%)
Abscess drainage	2	1	1
Hartmann procedure	1	0	1
Redo anastomosis	1	0	1
Stricureplasty	1	1	0
Debridement	1	1	0
Non surgical complications	2	0	2
Interventional radiology (abscess drainage)	1	0	1
Gastroscopy	1	0	1

Fifteen patients had post-operative complications (30.6%). Four patients sustained prolonged ileus (> 7 days); all were managed conservatively. Two patients in the repair group (who had been converted to open surgery) had surgical site infection (SSI) that was managed conservatively. One patient in the repair group had a deep organ/space intraabdominal infection (OS/SSI) vs. eight (29.6%) in the control group. Immediate outcome of the re-laparoscopic operation with anastomotic preservation and additional interventions are indicated in Tables 3 and 4.

One patient developed a short (< 1 cm) anastomotic stenosis 1 month after stoma closure which was managed by dilation as an outpatient. One patient developed stricture 2 months after transanal endoluminal repair and was managed by strictureplasty. Intra-operative bowel serosa tear occurred during adhesiolysis in two patients in the repair group: both were repaired immediately. In the control group, one patient had gastric ulcer and two patients sustained atelectasis. One patient died due to sepsis 62 days after the second operation.

Three patients (13.6%) in the repair group required an additional intervention compared to five (18.5%) in the control group but the difference was not statistically significant. The operations are indicated in Table 4.

Outcomes were uneventful in all eight patients (18.6%) who required an additional intervention.

Four patients (18.2%) in the repair group had a permanent stoma. The reasons why the stoma was not reversed were poor performance status and cancer progression in two patients each. Seven patients (25.9%) in the control group did not have stoma reversal: the reasons were poor performance status in 3, persistent purulent discharge (including the patient who died) and cancer progression in two patients each. There were no other complications related to stoma closure. No anastomotic sinus, fistula, or recurrence was noted at 3-year follow up.

## Discussion

Our study found that laparoscopic reintervention with anastomotic preservation for the management of colorectal anastomotic leakage was a viable option in 49 patients, thanks to early revisional surgery. The overall morbidity rate was 30.6% and one patient died (mortality rate 2%). Combined laparoscopy and transanal endoluminal repair of anastomotic leakage was associated with a lower overall complication rate (13.6% vs. 44.4%, p = 0.03) and fewer deep/organ space intraabdominal infections (4.5% vs. 29.6%, p = 0.03) compared with the control group. Also, a lower permanent stoma rate was noted in the repair group compared to the no repair group (18.2% vs. 25.9%, p = 0.73).

In the absence of consensus on standardized management of colorectal anastomosis leakage, some authors would argue that patients with stoma or localized abscess should been managed conservatively or undergo percutaneous drainage without reoperation [15, 16]. However, definitive closure of leaks with these methods can take several weeks, or be complicated with sinus formation or fistula [17, 18]. Laparoscopic reintervention for anastomotic leakage after laparoscopic colorectal surgery has gained widespread acceptance and several reports have shown that it is safe [13, 15, 19–22]. Traditionally, management for anastomotic leakage was via laparotomy because of the fear of bowel injury due to distended bowel and/or inadequate exposure through the laparoscopic approach [13]. In our series, two patients had serosal injury which were repaired immediately; both patients had undergone late reintervention (> 5 days), that is recognized as a cause of bowel distension, dense adhesions and inadequate visualization [13]. This is an argument in favor of early reintervention. Compared to relaparotomy, laparoscopic reintervention has the advantage of reduced wound complications (infection and incisional hernia) [15, 19, 20] and maintains the advantages of laparoscopic surgery [23]. Indeed, as in our series, the previous trocar wounds can be reused for laparoscopic reintervention and the minilaparotomy wound does not have to be re-opened. There were three patients who had surgical site infection and three patients with ventral hernia in our series. All six patients with wound complications were patients who had undergone conversion.

Another concern of laparoscopic reintervention for leaks has been that pneumoperitoneum would cause fecal ascites and then exacerbate intra-abdomen infection [21]. However, several studies have shown that laparoscopy does not increase intra-abdomen infection when compared with laparotomy [16, 20]. Lee et al. reported a 6.6% intra-abdominal infection rate after laparoscopic reintervention compared to 31.3% in the open group [20]. However, there was no clear definition of intra-abdomen infection or the additional interventions related to intra-abdomen infection. Although intraabdominal infection was the major complication after laparoscopic reintervention in our series, all patients had an uneventful recovery after either conservative therapy or additional intervention.

Most studies on laparoscopic reintervention for colorectal anastomosis leakage have recommended “divert and drain” with anastomosis preservation [13, 14, 19, 20]. Reports of colorectal anastomosis repair are rare [13, 20, 22]. Lee et al. reported 61 laparoscopic reinterventions for colorectal anastomosis leakage: 12 patients had trans anal repair [20]. However, the authors did not describe the outcome after anastomotic repair. Brunner et al. reported two patients who underwent trans anal repair with a single-port device (SILSTM Port CovidienTM); no stoma was performed [22] similar to our previously reported technique [13].

Our overall permanent stoma rate was 20.4% (10/49), similar to other studies [14, 16, 20]. This includes both the patients with stoma formation at the original operation (created according to patient status and surgeon preferences) and those who received a stoma at the reoperation. Of note, although there were fewer patients with permanent stoma in the repair group (18.2% vs. 25.9%), the difference was not statistically significant. When complicated by sinus formation and/or fistula, diverting stoma and simple drainage without repair may lead to delayed or no stoma closure [17]. None of our patients had sinus formation or late fistula. Furthermore, intestinal healing after anastomotic leak can be associated with intense fibrosis and eventually some degree of anastomotic stricture [24, 25]. We believe that early repair could possibly avoid these complications or at least reduce the inflammatory response associated with their persistence. The improved post-operative course with fewer intra-operative complications may explain why more patients in our series were scheduled for stoma reversal. However, our numbers are small and long term follow-up is needed.

Our morbidity rate (30.6%) is at the lower limit of the range reported in the literature [19, 25, 26]. However, none of these studies concerned the outcome of transanal endoluminal repair. Morbidity in the control (no-repair) group was high (13.6% vs. 44.4%, p = 0.03) although there was no difference in APACHE II score (7.9 vs. 10.3, p = 0.12), anastomotic defect characteristics, or diffuse peritoneal contamination (27.3% vs. 37.1%, p = 0.47) between the two groups. Possible explanations include the higher deep O/S SSI rate in the control group (29.6% vs. 4.5%, p = 0.03). When the anastomotic site is not repaired, it is possible that the leaking site could still be an active source of infection, even though the leak will eventually heal. Endoscopic vacuum-assisted closure has been reported as viable option to deal with anastomotic leak after low anterior resection with effective control of septic focus [27]. However, the duration of treatment can be long (34.4 ± 19.4 days) [27]. Although we have no formal proof, we believe that the ease with which the leak can be assessed and repaired endo-luminally (vs. trans abdominal laparotomy or laparoscopy) are strong arguments in favor of the transanal endoluminal route for extraperitoneal colorectal anastomosis leakage repair and could decrease the intraabdominal infection rate associated with laparoscopic reintervention. Indeed, intraabdominal infection is the main complication after laparoscopic reintervention and it is also the main reason for additional interventions after reoperation [19, 20]. In our study, all patients who had O/S SSI (including four patients after additional intervention) could be managed safely by antibiotics, radiological drainage or re-laparoscopy. There was no statistically significant difference in additional intervention rates, hospital stay or outcomes between two groups.

Our study has several limitations. This study was retrospective, and the sample size was small (n = 49). Although there were variations in the stage of disease stage and the distance of the anastomosis from the anal verge, these differences were not statistically significant. As well, there was no statistically significant difference in outcome between the two groups in spite of these variations as well as to whether leakage occurred early or late, or the technique of anastomosis at primary surgery. In addition, the indication for transanal endoluminal repair and stoma creation was made at the surgeon’s discretion. Although it may be argued that many patients with anastomotic leak after anterior resection may be suitable for conservative treatment, this may prolong hospital stay, delay reintervention or compromise stoma reversal. According to our experience, re-laparoscopy is an early diagnostic tool for anastomosis leakage and transanal endoluminal repair is easier when re-operation is early [12, 13] because bowel tissues become more inflamed, and adhesions are firmer when the intervention is performed at a later stage.

## Conclusions

This series confirms that laparoscopic reintervention with anastomotic preservation is safe and feasible for anastomotic leakage after low anterior resection. Furthermore, transanal endoluminal repair is effective in the management of colorectal anastomosis leakage under specific circumstances. Compared to controls (no repair), transanal endoluminal repair was associated with lower morbidity and in particular, OS/ISS; the permanent stoma rate was lower. However, our sample size was small and these promising results need to be reproduced in larger and ideally randomized studies comparing transanal endoluminal anastomotic repair to drainage (with or without stoma).

## Data Availability

Data sharing is not applicable to this article as no datasets were generated or analyzed.
